# Unhealthy Alcohol Use, HIV Infection and Risk of Liver Fibrosis in Drug Users with Hepatitis C

**DOI:** 10.1371/journal.pone.0046810

**Published:** 2012-10-09

**Authors:** Roberto Muga, Arantza Sanvisens, Daniel Fuster, Jordi Tor, Elisenda Martínez, Santiago Pérez-Hoyos, Alvaro Muñoz

**Affiliations:** 1 Department of Internal Medicine, Hospital Universitari Germans Trias i Pujol, Universitat Autònoma Barcelona, Barcelona, Spain; 2 Section of General Internal Medicine, Boston Medical Center, Boston University School of Medicine, Boston, Massachusetts, United States of America; 3 Department of Public Health, Institut de Recerca Hospital Vall d’Hebrón, Universitat Autònoma Barcelona, Barcelona, Spain; 4 Department of Epidemiology, Johns Hopkins University, Bloomberg School of Public Health, Baltimore, Maryland, United States of America; National Institute of Allergy and Infectious Diseases, United States of America

## Abstract

**Aim:**

To analyze alcohol use, clinical data and laboratory parameters that may affect FIB-4, an index for measuring liver fibrosis, in HCV-monoinfected and HCV/HIV-coinfected drug users.

**Patients and Methods:**

Patients admitted for substance abuse treatment between 1994 and 2006 were studied. Socio-demographic data, alcohol and drug use characteristics and clinical variables were obtained through hospital records. Blood samples for biochemistry, liver function tests, CD4 cell count, and serology of HIV and HCV infection were collected at admission. Multivariate linear regression was used to analyze the predictors of FIB-4 increase.

**Results:**

A total of 472 (83% M, 17% F) patients were eligible. The median age at admission was 31 years (Interquartile range (IQR) 27–35 years), and the median duration of drug use was 10 years (IQR 5.5–15 years). Unhealthy drinking (>50 grams/day) was reported in 32% of the patients. The FIB-4 scores were significantly greater in the HCV/HIV-coinfected patients (1.14, IQR 0.76–1.87) than in the HCV-monoinfected patients (0.75, IQR 0.56–1.11) (p<0.001). In the multivariate analysis, unhealthy drinking (p = 0.034), lower total cholesterol (p = 0.042), serum albumin (p<0.001), higher GGT (p<0.001) and a longer duration of addiction (p = 0.005) were independently associated with higher FIB-4 scores in the HCV-monoinfected drug users. The effect of unhealthy drinking on FIB-4 scores disappeared in the HCV/HIV-coinfected patients, whereas lower serum albumin (p<0.001), a lower CD4 cell count (p = 0.006), higher total bilirubin (p<0.001) and a longer drug addiction duration (p<0.001) were significantly associated with higher FIB-4 values.

**Conclusions:**

Unhealthy alcohol use in the HCV-monoinfected patients and HIV-related immunodeficiency in the HCV/HIV-coinfected patients are important risk factors associated with liver fibrosis in the respective populations

## Introduction

Liver fibrosis is the main predictor of whether chronic hepatitis C will progress to cirrhosis and end-stage liver disease [1]. Because the complications of liver disease mainly occur in patients with advanced-stage fibrosis, assessing chronic hepatitis C early is essential when evaluating at-risk patients [2]. In Western countries, more than 50% of new HCV infections are associated with drug abuse. However, this particular population also has lower rates of clinical assessment and chronic hepatitis C treatment. Given the likelihood of new and more effective treatments, drug abusers with chronic hepatitis C would benefit from simple, non-invasive measurements of liver fibrosis.

The cofactors associated with chronic hepatitis C progression differ among studies; alcohol abuse, male gender, age at infection, body mass index, and coinfection with human immunodeficiency virus infection (HIV) and Hepatitis B virus infection (HBV) have been related to more rapid disease progression [1]–[5]. In HCV/HIV-coinfected individuals, CD4 cell counts below 200 cells/µL have been associated with liver fibrosis progression [6]. In parallel, highly active antiretroviral therapy (HAART) has been shown to reduce liver-related deaths [7], [8].

In HIV-negative patients, it is well established that alcohol abuse and HCV infection have a synergistic effect on liver fibrosis. However, there are conflicting results regarding the independent effect of alcohol on liver damage in HCV/HIV-coinfected patients [6], [9], [10].

Liver biopsy is the gold standard for assessing fibrosis [11]. However, assessing liver disease through an invasive procedure is unlikely in patients with substance abuse [12]. Furthermore, eligibility for chronic hepatitis C treatment in this population is low compared with eligibility in other populations [13], [14]. To a certain extent, the evolution of liver disease in drug abusers parallels the natural history of chronic hepatitis C.

Several non-invasive markers of liver fibrosis have been proposed as alternatives to liver biopsy. Some of these markers reflect the modified extracellular matrix turnover that occurs during fibrogenesis [15], [16], whereas others reflect alterations in hepatic function [17], [18]. FIB-4 was initially described in 2006 [18], and since then, it has been proposed as reliable marker of fibrosis in both HCV-monoinfected and HCV/HIV-coinfected individuals [18], [19]. FIB-4 correlates well with liver biopsy in patients with and without advanced fibrosis [20], [21]. Moreover, non-invasive markers of liver fibrosis have been proposed as predictors of all-cause and liver-related mortality [22], [23].

Although abuse of alcohol and illegal drugs is frequent in patients with HIV infection and HCV infection, it is unclear how non-invasive liver fibrosis tests may reflect disease progression. In this study, we hypothesize that certain clinical and laboratory characteristics may influence a simple index of fibrosis and that the cofactors associated with elevated FIB-4 scores may differ between HCV-monoinfected patients and HCV/HIV-coinfected patients. Hence, the primary objective of the study was to characterize the putative differences in risk factors for elevated liver function biomarkers between HCV-monoinfected and HCV/HIV-coinfected patients.

## Patients and Methods

### Study Population

This was a cross-sectional study of patients admitted for substance abuse treatment between 1994 and 2006. The demographic and drug use characteristics were recorded through a structured questionnaire administered by a physician the day of admission. Questions related to drug and alcohol abuse included: (i) the main drug of abuse (type of drug, age at first use, duration of drug use and route of administration), (ii) poly-drug use (yes/no) (iii) alcohol consumption: do you regularly drink alcohol? (yes/no); if yes, do you drink 5 or more standard drinks per day?. A standard drink unit contains 12–14 grams of alcohol and unhealthy alcohol consumption was defined as a daily alcohol intake ≥50 grams (g) [24], [25] in the 6-month period prior to admission. All participants gave written informed consent. The methods used in this study complied with the ethical standards for medical research and principles of good clinical practice defined by the World Medical Association’s Declaration of Helsinki. The study was approved by the Ethics Committee of the Hospital Universitari Germans Trias i Pujol.

Routine laboratory parameters, including liver function tests and serology for HIV infection and HCV infection, were analyzed at admission. Other characteristics of admission for substance abuse treatment have been described elsewhere [26].

The liver function tests and biochemical parameters were assessed using an Olympus 5200 Multichannel chemistry analyzer. The procedure, which remained the same throughout the study, was based on the reference method recommended by the International Federation of Clinical Chemistry.

HIV infection was identified by an enzyme-linked immunosorbent assay. Repeatedly reactive samples were confirmed by the Western immunoblot technique.

HCV infection was assessed prior to or during admission by a second- or later-generation enzyme immunoassay (Ortho Diagnostics, Raritan, NJ). The positive samples were confirmed by either a recombinant immunoblot assay (RIBA HCV 2 SIA, Chiron Corporation, Emeryville, CA) or a qualitative/quantitative assay (COBAS AMPLICOR, Roche Diagnostic Systems, Branchburg, NJ).

### Outcome

The primary outcome was the FIB-4 score, which was calculated as
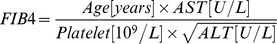



FIB-4 scores lower than 1.45 indicate lack of liver fibrosis with a negative predictive value of 90% and a sensitivity of 70% [18]. FIB-4 scores greater than 3.25 indicate significant liver fibrosis with a positive predictive value of 65% and a specificity of 97% [18].

### Statistical Analysis

All of the analyses were conducted separately for the HCV-monoinfected (N = 228) and the HCV/HIV-coinfected (N = 244) individuals. We used medians and interquartile ranges (IQRs) to describe the quantitative variables and absolute frequencies and percentages to describe the qualitative variables.

The distribution of FIB-4 score was strongly skewed to the right (i.e., there were several very high values); we therefore normalized it for analysis purposes using a logarithmic transformation.

We used multiple linear regression models to determine the FIB-4 predictive values of the variables. There were three types of predictors: (1) binary, which included sex, alcohol use, and HBsAg; (2) continuous on a natural (additive) scale, which included body mass index (BMI), CD4 cell count, total cholesterol, alkaline phosphatase, and duration of drug use; and (3) continuous on a logarithmic (multiplicative) scale, which included total bilirubin, serum albumin, and GGT. The decision to analyze a variable using a logarithmic scale was based on the need to reduce the undue influence of high values in predictors with strong right skewness.

The interpretation of the regression coefficients differed among the three types of predictors. Specifically, the regression coefficients of the binary variables represented the percentage FIB-4 difference between those with and without the condition; the regression coefficients of the additive continuous variables represented the percentage FIB-4 difference associated with an unitary increase or decrease in the variables, and the regression coefficients of the multiplicative continuous variables represented the percentage FIB-4 difference associated with an increment or decrement in the variables.

The intercept represented the expected FIB-4 score in an individual with zero values for all of the predictors.

The test results were considered to be statistically significant if the resulting P-*value* was <.05. The statistical analysis was performed using the SPSS software, version 15.0.1 (SPSS, Chicago, IL, USA).

## Results

Patients were eligible for this study if they had chronic HCV infection (N = 544). Patients with aminotransferase levels 10 times greater than the upper limit of the normal range (N = 5,1.0%), patients who had received HCV antiviral therapy (N = 6,1.1%) and patients with antecedent of decompensated liver cirrhosis (N = 10,1.8%) were excluded. In addition, patients with an HCV-RNA level below the limit of detection (<50 IU/mL) were excluded (N = 9, 1.7%). Finally, patients with outlier laboratory values and those with incomplete data for calculating FIB-4 score were also excluded (N = 42, 7.7%). After these exclusions, the study population consisted of 472 patients and 244 patients (52%) were coinfected with HIV. Table 1 shows the descriptive statistics at admission for the entire group and for the HCV-monoinfected (N = 228) and HCV/HIV-coinfected (N = 244) subgroups. Overall, 17% of the patients were women, the median age at admission was 31 years (IQR 27–35 years), the median BMI was 22 kg/m^2^, the median duration of drug use was 10 years, and unhealthy drinking was reported in 32% of the patients. In addition to decreased CD4 cell counts, the HCV/HIV-coinfected patients had a longer median drug use duration, an increased frequency of unhealthy alcohol intake, lower levels of total cholesterol, higher levels of GGT and a higher prevalence of HBsAg than those infected with HCV only. The median AST and ALT levels and platelet counts were 35 U/L, 47 U/L, and 180 × 10^9^/L, respectively.

**Table 1:** Descriptive statistics (median [IQR] or n (%)) of HCV-monoinfected and HCV/HIV-coinfected patients at admission to substance abuse treatment.

	HCV	HCV/HIV		Total
	N = 228	N = 244	p_value*	N = 472
*Socio-demographic and anthropometric*				
**Females**	35 (15.4%)	45 (18.4%)	0.371	80 (16.9%)
**Age, years**	30 [26], [34]	31.5 [28, 35]	0.008	31 [27, 35]
**Body mass index, kg/m^2^ (N = 421)**	22.3 [20.7, 24.6]	21.6 [19.6, 23.6]	0.000	21.9 [20.2, 23.9]
*Drug use*				
**Unhealthy alcohol use (N = 440 )**	62 (28.2%)	79 (35.9%)	0.082	141 (32.0%)
**Duration of drug use, years (N = 463)**	7.6 [3.5, 12.0]	12.0 [7.6, 16.0]	0.000	10.0 [5.5, 15.0]
*Laboratory parameters*				
**Hepatitis B surface Antigen (N = 440 )**	9 (4.2%)	15 (6.7%)	0.243	24 (5.5%)
**Total cholesterol, mg/dL**	162 [143, 178]	149 [128, 170]	0.001	155 [135, 174]
**Alkaline Phosphatase, U/L**	70 [55, 82]	70 [59, 86]	2.664	70 [56, 84]
**Total bilirubin, mg/dL**	0.4 [0.3, 0.6]	0.4 [0.3, 0.6]	0.297	0.4 [0.3, 0.6]
**Albumin, g/L (N = 438)**	39 [36, 42]	38 [35, 41]	0.013	39 [36, 41]
**GGT, U/L**	33 [19, 62]	44.5 [24, 93]	0.052	38 [22, 76]
**CD4 lymphocytes, cells/**µ**L (N = 456)**	1225 [933, 1428]	383 [204, 661]	0.000	742 [350, 1225]
*Laboratory components of FIB-4*				
**Platelets, 10^9^/L**	197 [166, 243]	163 [127, 196]	0.000	180 [146, 224]
**AST, U/L**	33.5 [21.0, 61.0]	37.5 [24.2, 61.0]	0.907	35.0 [23.0, 61.0]
**ALT, U/L**	54.0 [23.0, 98.0]	43.0 [25.0, 71.0]	0.001	47.0 [24.0, 84.0]

* p value for the comparison between HCV-monoinfected and HCV/HIV-coinfected patients; p values correspond to χ square test in categorical variables and t test for differences of mean values in continuous variables.

Thirty-one percent of the HIV-positive patients were receiving antiretroviral therapy at admission, and 48% had never received antiretroviral therapy.

**Figure 1:**
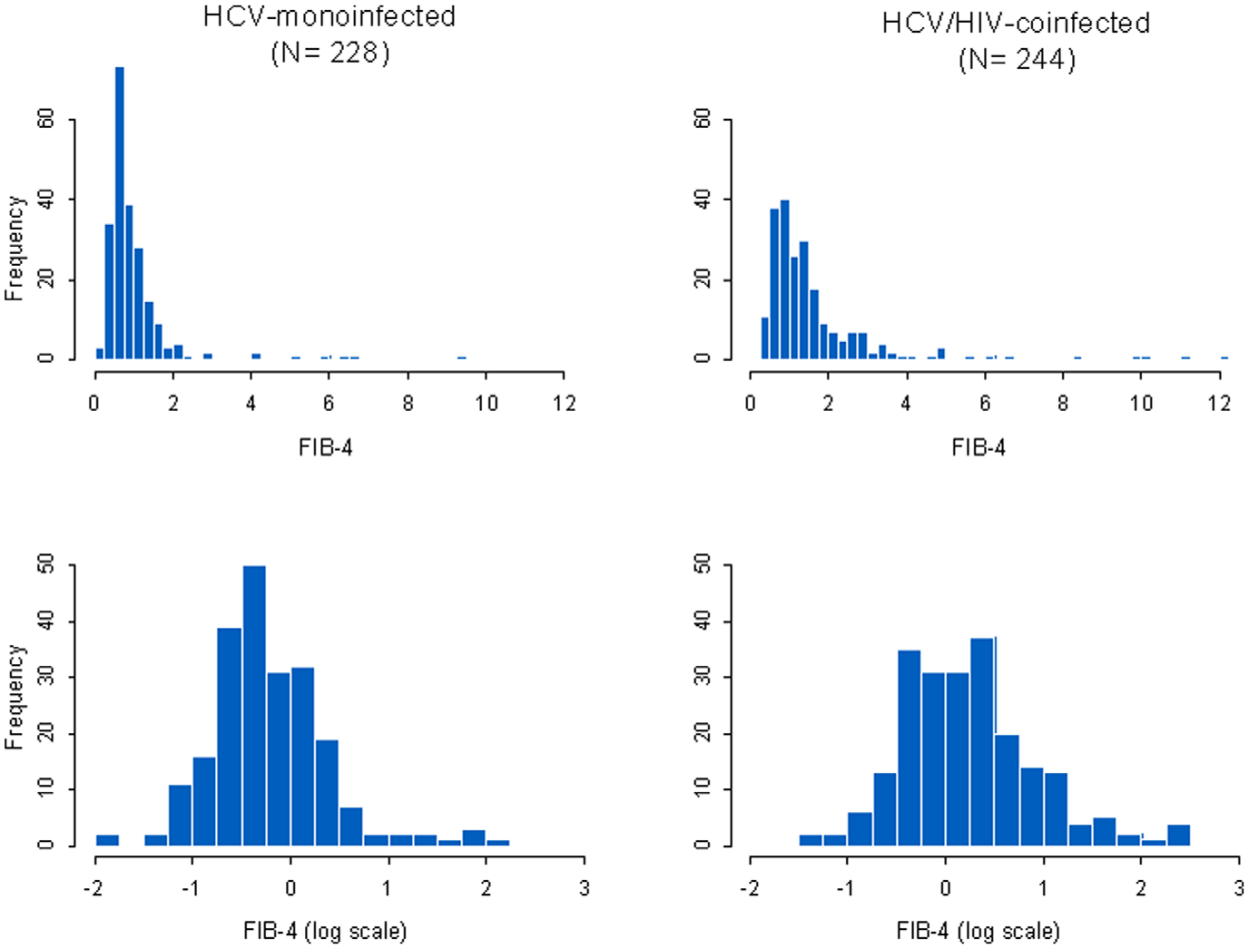
Distribution of FIB-4 score and log FIB-4 score according to HCV-monoinfection and HCV/HIV-coinfection.

The median FIB-4 score at admission was 0.93 (IQR 0.65–1.46); it was significantly higher in the HCV/HIV-coinfected patients (1.14, IQR 0.76–1.87) than in the HCV-monoinfected patients (0.75, IQR 0.56–1.11). Figure 1 shows the distribution of the FIB-4 scores in the two groups on both natural and logarithmic scales. As can be seen in the bottom panels of Figure 1, the log-transformed FIB-4 scores approximately followed a normal distribution, rendering normally based methods appropriate for the analysis.

### Regression Analysis of FIB-4 (Log Scale)

We conducted univariate regressions of the variables shown in Table 1 against the log-transformed FIB-4 scores; only the variables that define FIB-4 were not used in the univariate regressions. The two columns with univariate headings in Table 2 show the results of the univariate analyses separately for the monoinfected and coinfected patients.

**Table 2:** Percentage change in FIB-4 score associated with differences in predictors of higher FIB-4 scores.

	HCV-monoinfected		HCV/HIV-coinfected	
	N = 228		N = 244	
	Univariate	Multivariate	Univariate	Multivariate
**Variable**	**% (p_value)**	**% (p_value)**	**% (p_value)**	**% (p_value)**
**Intercept**	NA	0.778*	NA	0.875**
		(95% CI : 0.705, 0.861)		(95% CI: 0.762, 1.005)
**Female to Male**	−11.6% (0.266)		+11.5% (0.343)	
**Unhealthy alcohol use**	**+46.7% (<.001)**	**+20.6% (0.034)**	+3.9% (0.695)	
**HBsAg positive**	+25.1% (0.268)		+31.3% (0.155)	
**BMI: increase of 1 kg/m^2^**	**+4.3% (0.001)**	+2.2% (0.069)	+0.8% (0.609)	
**Duration of drug use: increase of 5 years**	**+9.4% (0.007)**	**+10.0% (0.005)**	**+21.5% (<.001)**	**+13.9% (<.001)**
**Cholesterol: decrease of 20 mg/100 mL**	**+5.4% (0.018)**	**+4.5% (0.042)**	**+6.6% (0.006)**	+2.4% (0.318)
**Alkaline Phosphatase: increase of 10 U/L**	+2.7% (0.108)		**+4.9% (0.001)**	+0.9% (0.618)
**CD4: decrease of 100 cells/**µL	NA	NA	**+5.2% (<.001)**	**+3.6% (0.007)**
**Bilirubin: 1.5-fold increment**	**+12.7% (<.001)**	+5.7% (0.097)	**+22.8% (<.001)**	**+22.2% (<.001)**
**Albumin: 1.1-fold decrement**	**+19.8% (<.001)**	**+19.1% (<.001)**	**+15.4% (<.001)**	**+12.9% (<.001)**
**GGT: 2-fold increment**	**+20.3% (<.001)**	**+12.3% (0.001)**	**+16.4% (<.001)**	+7.3% (0.061)

* Expected value of FIB-4 for individuals with predictors at BMI = 22 kg/m^2^, no alcohol consumption, total cholesterol = 155 mg/100 mL, Total bilirubin = 0.4 mg/dL, albumin = 39 g/L, GGT = 38 U/L, and duration of IDU = 10 years.

** Expected value of FIB-4 for individuals with predictors at CD4 = 900 cells/µL, total cholesterol = 155 mg/100 mL, total bilirubin = 0.4 mg/dL, albumin = 39 g/L, Alkaline Phosphatase = 70 U/L, GGT = 38 U/L, and duration of IDU = 10 years.

In the univariate models for the HCV-monoinfected patients, unhealthy alcohol use, higher BMI, longer duration of drug use, lower cholesterol, higher bilirubin, lower albumin, and higher GGT were significantly associated (p<0.05) with higher FIB-4 scores. In the coinfected patients, higher FIB-4 scores were found to be significantly associated (p<0.05) with a longer drug use duration, lower cholesterol, higher alkaline phosphatase, lower CD4 cell count, higher bilirubin, lower albumin, and higher GGT.

For each group, the variables that showed significant relationships in the univariate analyses were used in a multivariate model. In the multivariate model for the HCV-monoinfected patients, unhealthy alcohol use (p = 0.034), longer drug use duration (p = 0.005), lower cholesterol (p = 0.042), lower albumin (p<0.001), and higher GGT (p = 0.001) continued to be significantly associated with higher FIB-4 scores.

In the coinfected patients, longer drug use duration (p<0.001), lower CD4 cell count (p = 0.007), higher bilirubin (p<0.001), and lower albumin (p<0.001) were significantly associated with higher FIB-4 scores.

Longer drug use duration and lower albumin levels were significantly associated with increased FIB-4 scores in both groups. By contrast, unhealthy alcohol use was strongly predictive of high FIB-4 scores only in the HCV-monoinfected group; similarly, high total bilirubin levels were associated with higher FIB-4 scores only in the coinfected patients.

Although the primary aim of the study was the characterization of the variables that were associated with FIB-4 increase in HCV-monoinfected and HCV/HIV-coinfected, the intercepts of the multivariate models provide a means of comparing a hypothetical HCV-monoinfected individual with a hypothetical HCV/HIV-coinfected individual, assuming that both have 900 CD4 cells/µL and that all of the other variables are equal. The slightly increased FIB-4 intercept value (0.778 in the monoinfected and 0.875 in the coinfected patients) was not statistically significant (p = 0.218). However, for each decline of 100 CD4 cells/µL among the coinfected patients, there was a significant FIB-4 increase of 3.6% (p = 0.007) (Table 2).

**Figure 2:**
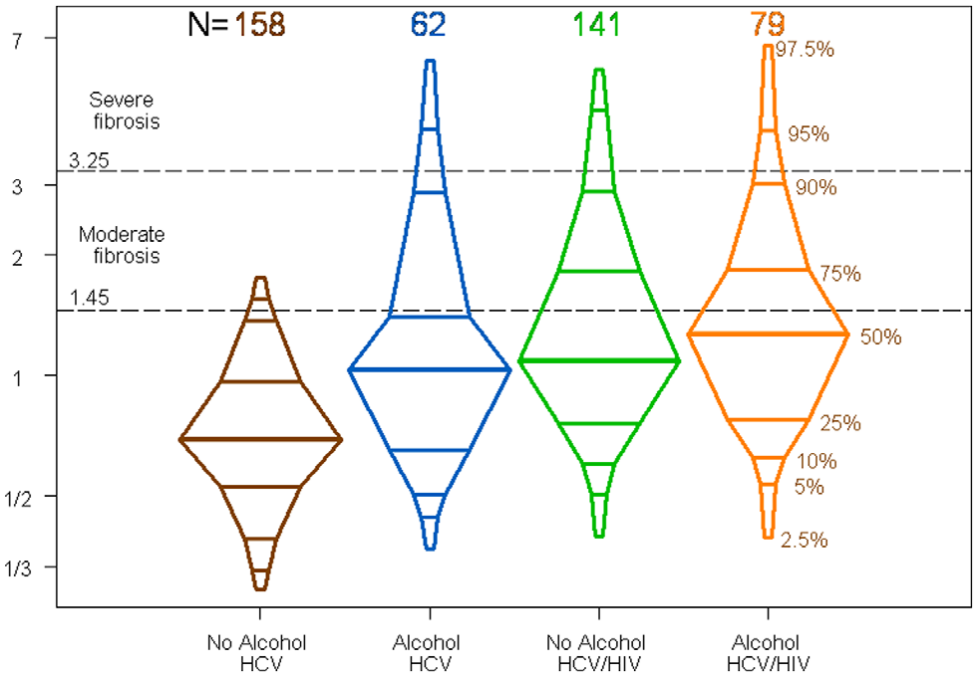
Distribution of FIB-4 scores according to unhealthy alcohol use in HCV-monoinfected and HCV/HIV-coinfected patients.

To further characterize the differential effect of unhealthy alcohol use on FIB-4 scores, Figure 2 shows the distributions of the FIB-4 scores in the four groups by alcohol consumption and HIV coinfection. Figure 2 shows box plots that are graphically enhanced to show the 2.5^th^, 5^th^, 10^th^, 25^th^, 50^th^, 75^th^, 90^th^, 95^th^ and 97.5^th^ percentiles of the FIB-4 distribution. It is clear from Figure 2 that the primary difference was between the non-drinking HCV-monoinfected patients and the other three groups. In particular, the increased FIB-4 score due to unhealthy alcohol use among the HCV-monoinfected patients was similar to the effect of HIV-related immunodeficiency among the non-drinkers. The additional increase in FIB-4 among the coinfected individuals with unhealthy alcohol use was small and not significant (p = 0.695).

## Discussion

Individuals with histories of drug use account for the majority of new hepatitis C infections in Western countries. This population is at risk for liver fibrosis, and a number of disease progression cofactors highlight the relevance of medical assessment. Evaluating liver fibrosis via non-invasive tests early in the course of drug addiction may increase the proportion of patients who are eligible for treatment. This study of young adults with chronic hepatitis C shows that the factors associated with higher FIB-4 scores clearly differed between the HCV-monoinfected and HCV/HIV-coinfected individuals.

The main contribution of the study is related to the fact that unhealthy alcohol use had a differential effect on FIB-4 values if patients have hepatitis C alone or HCV/HIV coinfection. Unhealthy alcohol drinking has been regarded as a major contributor to the progression of liver disease in the setting of chronic hepatitis C [27] and, a synergistic effect between HCV and alcohol has been proposed [28]. However, even though alcohol use is among the cofactors related with liver fibrosis in coinfection in studies with liver biopsy [6], [29], the present and other studies that have used non- invasive methods to estimate fibrosis [9], [10], does not detect an additional effect of alcohol drinking on the FIB-4 of HCV/HIV-coinfected patients.

In coinfected patients with unhealthy alcohol consumption, the FIB-4 does not reflect the negative impact of alcohol intake on liver fibrosis. Therefore, clinicians may not be able to assess the impact of ethanol nor can advise the patient on the risk of disease progression. On the contrary, unhealthy alcohol use is reflected in the FIB-4 of the monoinfected patients thus making possible preventive interventions to reduce harm.

Unhealthy alcohol use in the HCV-monoinfected patients and HIV-related immunodeficiency in the HCV/HIV-coinfected patients are the most important cofactors associated with fibrosis progression in the respective populations. In addition, we found that drug use duration and serum albumin were correlated with the FIB-4 scores of both the monoinfected and coinfected patients, whereas unhealthy alcohol use, GGT and total cholesterol were associated with higher FIB-4 scores only in the monoinfected patients. The effect of HIV-related immunodeficiency in the coinfected patients was strong (an increase of 3.5% in the FIB-4 score for every 100 CD4 cells/µL decrease). Furthermore, we did not observe differing FIB-4 values between the HCV-monoinfected and coinfected individuals with CD4 cell counts above 900 cells/µL. This observation suggests that FIB-4 elevation is associated with immunoactivation and the resulting decrease of CD4 cell counts in HCV/HIV-coinfected drug users.

The relationship between HIV-related immunodeficiency and liver fibrosis progression has been described in coinfected patients [6], [29], [30]; in fact, treating HIV/AIDS with HAART has been shown to reverse the effect of HIV-related immunodeficiency in patients with chronic hepatitis C [7], [26], [29], [31].

In this study, decreased serum albumin and increased total bilirubin were associated with elevated FIB-4 scores. This finding may facilitate identifying a subpopulation of patients at increased risk for cirrhosis. It is well known that albumin and bilirubin are key components of the Child-Turcotte-Pugh score that clinicians use to assess decompensated liver cirrhosis.

In individuals with history of injection drug use, the duration of injection use is a surrogate for the duration of HCV infection [32]. As expected, the duration of drug addiction in this study was related to increased FIB-4 scores.

It has been reported that HCV infection itself lowers both low-density lipoprotein (LDL) and total cholesterol and that patients treated for chronic hepatitis C had larger increases in LDL and total cholesterol from baseline [33]. Interestingly, the current study did not find a significant association between cholesterol levels and FIB-4 scores among the HCV/HIV-coinfected patients.

This study has a number of limitations that should be mentioned. First, the alcohol intake assessment was limited to one categorical variable (>50 g/day, ≤50 g/day in the 6-month period before admission), and there was no information on the history and complications of alcohol consumption. In previous studies, however, recent alcohol consumption has been treated as a dichotomous variable using a threshold of 40–50 grams of ethanol per day or using the definition of heavy alcohol intake provided by the US National Institute on Alcohol Abuse and Alcoholism (NIAAA) [6], [9], [10]. Second, we used a single measurement of laboratory parameters to calculate the FIB-4 scores which precluded examining the evolution of fibrosis over time. Furthermore, nearly half of the patients had normal aminotransferases values, as has been previously described for IDUs [34]; despite the lack of correlation between liver enzyme alterations and liver damage, it is possible that FIB-4 scores are affected by normal liver enzymes [20]. Third, the HAART status of the HIV-positive patients was represented by a qualitative covariate, which hindered an analysis of the effect of antiretroviral treatment on FIB-4.

In summary, this study shows that unhealthy alcohol use strongly influence FIB-4 in HCV- monoinfected patients, whereas in the context of HCV/HIV coinfection, HIV-related immune depression exerts a major negative role on FIB-4 results, with no significant worsening by alcohol intake.
